# Simvastatin Attenuates the Oxidative Stress, Endothelial Thrombogenicity and the Inducibility of Atrial Fibrillation in a Rat Model of Ischemic Heart Failure

**DOI:** 10.3390/ijms150814803

**Published:** 2014-08-22

**Authors:** Kyoung-Im Cho, Sang-Ho Koo, Tae-Joon Cha, Jung-Ho Heo, Hyun-Su Kim, Gee-Bum Jo, Jae-Woo Lee

**Affiliations:** Division of Cardiology, Department of Internal Medicine, Kosin University College of Medicine, Busan 602702, Korea; E-Mails: kyoungim74@gmail.com (K.-I.C.); paulkoo@hanmail.net (S.-H.K.); lduggymdc@gmail.com (J.-H.H.); libaek@gmail.com (H.-S.K.); kyoungim74@dreamwiz.com (G.-B.J.); jwlee@ns.kosinmed.or.kr (J.-W.L.)

**Keywords:** statin, atrial fibrillation, nitric oxide synthase, heart failure

## Abstract

Increased atrial oxidative stress has an important role in inducing and maintaining atrial fibrillation (AF), and the activation of the small GTPase Rac1 contributes to the oxidative stress. We investigated the relationship of Rac1, atrial endothelial thromboprotective markers and AF inducibility and if simvastatin has a potential beneficial effect on a myocardial infarction (MI)-induced heart failure (HF) rat model. Rats were randomized into three groups (shams, MI group and simvastatin treatment group) and underwent echocardiography, AF induction studies and left atrial (LA) fibrosis analysis. Atrial Rac 1, sodium calcium exchanger (*I*_NCX_), sarcoplasmic reticulum calcium ATPase (SERCA), endothelial nitric oxide synthase (eNOS) and induced nitric oxide synthase (iNOS) were measured. AF inducibility, AF duration and LA fibrosis were significantly higher in the MI group (*p* < 0.001 *vs.* sham), which were significantly reduced by simvastatin (*p* < 0.05 *vs.* MI). The reduced expressions of atrial eNOS, SERCA, thrombomodulin, tissue factor pathway inhibitor and tissue plasminogen activator in the MI group were significantly improved by simvastatin. Furthermore, the increased expression of atrial iNOS, *I*_NCX_ and Rac1 activity were significantly decreased by the simvastatin. Oxidative stress, endothelial dysfunction and thrombogenicity are associated with the promotion of AF in a rat model of ischemic HF. These were associated with increased Rac1 activity, and simvastatin treatment prevents these changes.

## 1. Introduction

Atrial fibrillation (AF) is the most common clinically significant cardiac arrhythmia and is a potent risk factor for ischemic stroke [1]. Recent studies have suggested that inflammation and oxidative stress play a role in the pathogenesis of AF [2,3,4,5]. Because hypercholesterolemia reduces endothelial production and increases degradation of nitric oxide (NO) [6,7], cholesterol-lowering treatment by statins causes a significant improvement in endothelial function [6] by upregulating endothelial cell nitric oxide synthase (eNOS) and NO synthase [8]. Some studies have suggested that statins block atrial remodeling induced by substances such as NO and reactive oxygen species [9,10]. In addition to their indirect antiarrhythmic effects, statins may also exhibit direct antiarrhythmic effects by modulating the fatty acid composition and physiochemical properties of cell membranes, with resultant alterations in transmembrane ion channel properties [10,11]. Moreover, accumulating evidence suggests that statins may reduce vascular inflammation and the incidence of AF [12] by virtue of pleiotropic properties independent of lipid lowering and that statins may significantly reduce the incidence of AF in the setting of both primary and secondary prevention [13,14]. According to Sarr *et al.* [15], hydrophilic pravastatin exhibited the lowest association with the lipid monolayer, and lipophilic simvastatin showed a strong membrane elution ability, which can be explained by the hydrophobicity of the statin molecule [16]. These findings suggest that the difference of membrane permeability according to the lipophilicity might explain the impact of statin on the ion channel in the cell membrane.

Observations of ventricular cardiomyocytes in heart failure (HF) have revealed that the action potential duration is prolonged and that sarcoplasmic reticulum Ca^2+^ release is reduced under these conditions [17]. Abnormal intracellular Ca^2+^ cycling plays an important role in cardiac dysfunction and ventricular arrhythmogenesis in the setting of ischemic HF [18,19]. However, the antiarrhythmic effects of statins in ischemic HF are still unknown.

Atrial oxidative stress might play an important role in inducing and maintaining AF, and nicotinamide adenine dinucleotide phosphate (NADPH) oxidase activity is a major source of myocardial superoxide production [20]. Rac1 is necessary for the activation of the NADPH oxidase complex, and during activation, Rac1 binds guanosine triphosphate (GTP) and migrates to the membrane with the core cytosolic complex. Therefore, it has been suggested that the activation of Rac1 GTPase might contribute to the pathogenesis of AF via activation of superoxide producing NADPH oxidase [21].

We hypothesized that there would be the relationship between Rac 1 and AF inducibility, as well as atrial endothelial thromboprotective markers, which might be reduced by statin. Then, we hypothesized that the altered Ca^2+^ handling, inflammation and thromboprotective markers in ischemic HF are attenuated by the statin, which might explain the anti-arrhythmic role of statin in HF. Therefore, we investigated the changes in Rac 1, the inducibility of AF and atrial endothelial thromboprotective markers with or without statin in a rat model of ischemic HF model.

## 2. Results and Discussion

### 2.1. Echocardiographic Indices

**Figure 1:**
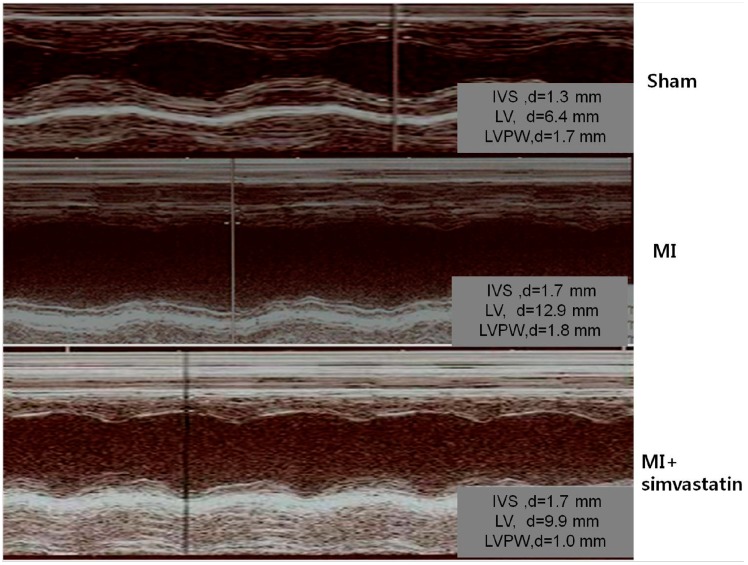
Echocardiograms exhibiting the left ventricular anteroseptal wall akinesia and dilated ventricular dimensions in myocardial infarction (MI) rats. These changes were slightly less pronounced in the simvastatin-treated group. IVS, interventricular septum; LV, left ventricle; LVPW, left ventricular posterior wall.

Echocardiographic parameter changes in each group are presented in Figure 1 and Table 1. In the myocardial infarction (MI) group (*n* = 10), the left ventricular end diastolic dimension and left atrial (LA) dimension were significantly increased and the ejection fraction significantly decreased (30% ± 1.6% *vs.* 83% ± 7.5%, *p* < 0.001) compared to the sham group (*n* = 10) (Figure 1, Table 1).

**Table 1:** Echocardiographic parameters.

Parameters	Sham ( *n* = 10)	MI ( *n* = 10)	MI-Simvastatin ( *n* = 10)
EF, %	83.0 ± 7.6	30.0 ± 16.1 *	43.0 ± 21.5 *
FS, %	48.5 ± 8.8	12.7 ± 7.5 *	20.6 ± 15.2 *
LVEDD, mm	7.9 ± 0.6	9.5 ± 1.3 **	9.6 ± 1.6 ***
LVESD, mm	4.0 ± 0.8	8.3 ± 1.5 *	7.8 ± 2.2 *
LAD, mm	3.4 ± 0.8	3.8 ± 0.9	3.6 ± 0.8

* *p* < 0.001 *vs.* sham; ** *p* < 0.01 *vs.* sham; *** *p* < 0.05 *vs.* sham; MI, myocardial infarction; EF, ejection fraction; FS, fractional shortening; LVEDD, left ventricular end diastolic dimension; LVESD, left ventricular end systolic dimension; LAD, left atrial dimension.

There was a significant increase in the heart weight in the MI group compared to the sham group (5.60 ± 0.22 *vs.* 3.37 ± 0.13 mg, *p* < 0.001). The MI + simvastatin group (*n* = 10) showed an increase in the ejection fraction (EF) (43.2% ± 2.2% *vs.* 30% ± 16%, *p* = 0.08) when compared to the MI group, but the difference was not statistically significant.

### 2.2. Atrial Fibrillation Induction Study

**Figure 2:**
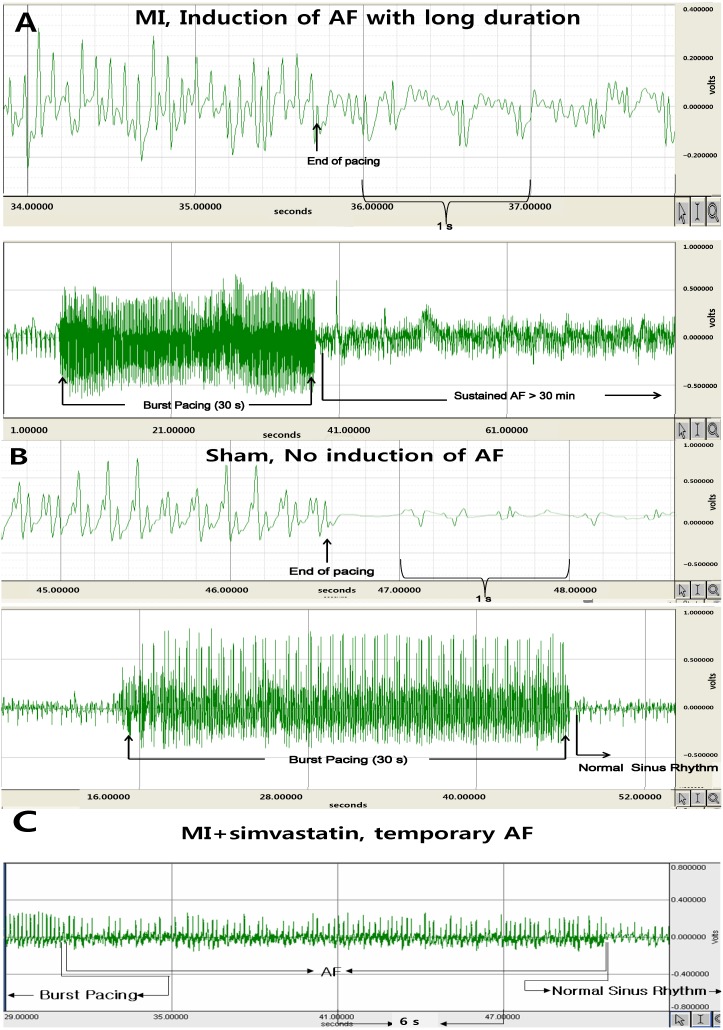
The electrocardiography shows the successful induction of atrial fibrillation (AF) following burst pacing in the myocardial infarction (MI group) (**A**); AF could not be induced in the sham group (**B**); and the duration of AF was lower in the MI + simvastatin group (**C**).

**Figure 3:**
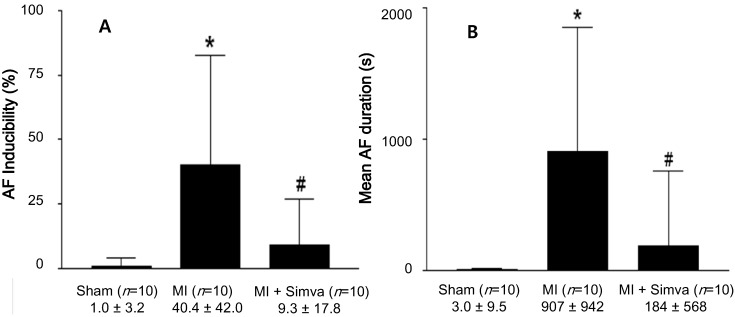
(**A**) The results of the atrial fibrillation (AF) induction studies. * *p* < 0.001 sham *vs.* myocardial infarction (MI), ^#^ *p* = 0.023 MI *vs.* MI + simvastatin; and (**B**) The mean duration of AF (seconds) following burst pacing. * *p* = 0.001 sham *vs.* MI and MI + simvastatin, ^#^ *p* = 0.031 MI *vs.* MI + simvastatin.

AF was induced more easily (40.4% ± 4.2% *vs.* 1.0% ± 3.2%, *p* < 0.001) in the MI group (*n* = 10) compared to the sham group (*n* = 10), but decreased after simvastatin treatment (*n* = 10, 9.3% ± 7.8% in the MI + simvastatin group, *p* < 0.05 *vs.* MI) (Figure 2 and Figure 3A). The duration of AF was also significantly increased in the MI group compared to the sham group (907 ± 942 *vs.* 3.0 ± 9.5 s, *p* < 0.001), but was moderately reduced after simvastatin treatment (184 ± 568 s in the MI + simvastatin group, *p* < 0.05 *vs.* MI) (Figure 3B).

### 2.3. Reduction of Atrial Fibrosis by Simvastatin

**Figure 4:**
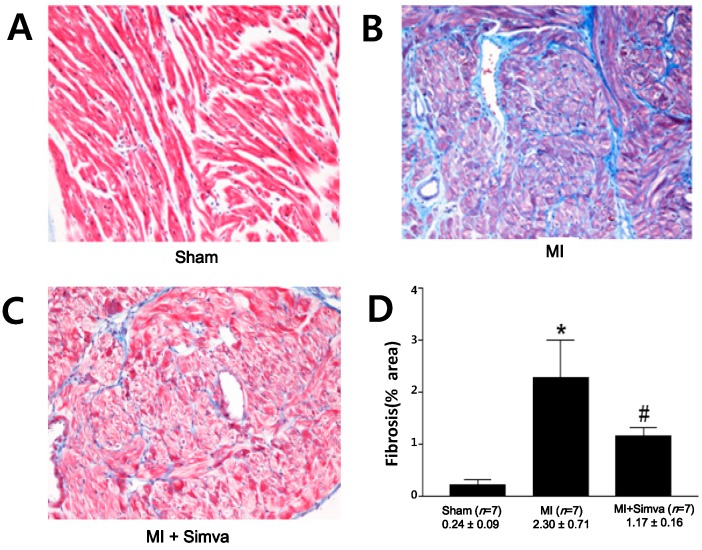
Masson’s trichrome staining of the left atrium. The amount of fibrosis was lower in the sham (**A**) than the MI group (**B**); but this increase was attenuated in the MI + simvastatin (**C**) group. Blue represents fibrosis. (**D**) The effect of simvastatin on fibrosis of the left atrium. * *p* = 0.001 sham *vs.* MI and MI + simvastatin, ^#^ *p* = 0.001 MI *vs.* MI + simvastatin.

The amount of fibrosis in the LA, as a percentage of perimuscular interstitial blue area except muscle for each rat, was significantly higher in the MI group than in the sham group (*n* = 7 for each group, 2.30% ± 0.71% *vs.* 0.24% ± 0.09%, *p* = 0.001) (Figure 4). The amount of fibrosis in the LA was significantly lower in the MI + simvastatin group than in the MI group (1.17% ± 0.16% * vs.* 2.30% ± 0.71%, *p* = 0.001).

### 2.4. Expression of Nitric Oxide Synthases and Calcium Handling Proteins

**Figure 5:**
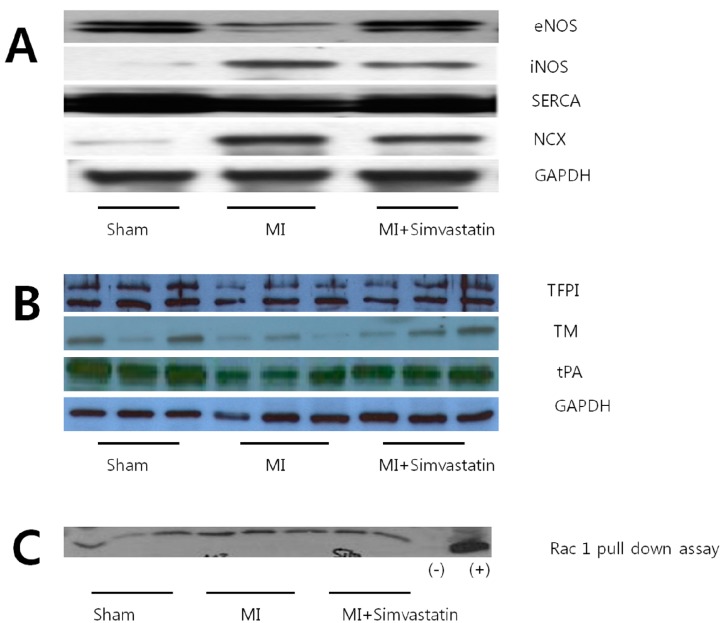
Alterations in eNOS, iNOS, SERCA, NCX (**A**), TFPI, TM, tPA (**B**) and Rac 1 (**C**) protein expression in the left atrium (eNOS, endothelial nitric oxide synthase; iNOS, induced nitric oxide synthase; NCX, sodium calcium exchanger; SERCA, sarcoplasmic reticulum calcium ATPase; TM, thrombomodulin; TFPI, tissue factor pathway inhibitor; tPA, tissue plasminogen activator; Rac 1, Rac 1 GTPase).

LA tissue levels of the eNOS (*p* < 0.05) and SERCA (*p* < 0.01) proteins were significantly lower in the MI group than in the sham group, but were significantly higher in the MI + simvastatin group than in the MI group (all *p* < 0.05, Figure 5A). The levels of iNOS and *I*_NCX_ proteins in LA tissue were significantly higher in the MI group than in the sham group (*p* < 0.01). The expression of iNOS and *I*_NCX_ was significantly lower in the MI + simvastatin group than in the MI group (*p* < 0.05) (Figure 5A and Table 2). All results were normalized to GAPDH data concomitantly obtained from the same samples.

**Table 2:** Densitometric analysis of western blot results.

Parameters	Sham ( *n* = 10)	MI ( *n* = 10)	MI-Simvastatin ( *n* = 10)
Fibrosis (%)	0.24 ± 0.09 ( *n* = 7)	2.30 ± 0.71 * ( *n* = 7)	1.17 ± 0.16 ^#^ ( *n* = 7)
eNOS	13.41 ± 2.58 ( *n* = 6)	6.75 ± 2.05 * ( *n* = 6)	12.06 ± 3.43 ^#^ ( *n* = 6)
iNOS	0.28 ± 0.30 ( *n* = 9)	7.33 ± 5.79 * ( *n* = 10)	1.60 ± 1.47 **^,#^ ( *n* = 12)
*I*_NCX_	0.79 ± 0.48 ( *n* = 8)	14.52 ± 11.87 * ( *n* = 11)	4.19 ± 11.87 *^,## ^( *n* = 12)
SERCA	30.90 ± 3.18 ( *n* = 6)	21.13 ± 1.36 * ( *n* = 6)	25.43 ± 3.27 **^,##^ ( *n* = 6)
TM	2.54 ± 0.47 ( *n* = 6)	0.80 ± 0.28 * ( *n* = 6)	1.85 ± 0.38 ^#^ ( *n* = 6)
TFPI	6.37 ± 0.71 ( *n* = 6)	3.15 ± 0.22 ( *n* = 6)	4.32 ± 0.47 ( *n* = 6)
tPA	2.73 ± 0.12 ( *n* = 6)	1.25 ± 0.12 ( *n* = 6)	2.01 ± 0.13 ( *n* = 6)
Rac 1	16,165 ± 1696 ( *n* = 6)	28,440 ± 1655 * ( *n* = 6)	19,275 ± 2236 ^#^ ( *n* = 5)

* *p* < 0.01 *vs.* sham, ** *p* < 0.05 *vs.* sham; ^#^ *p* < 0.001 *vs* MI + simvastatin; ^##^ *p* < 0.01 *vs.* MI + simvastatin; eNOS, endothelial nitric oxide synthase; iNOS, inducible nitric oxide synthase; NCX, sodium calcium exchanger; SERCA, sarcoplasmic reticulum Ca^2+^-ATPase; TM, thrombomodulin; TFPI, tissue factor pathway inhibitor; tPA, tissue plasminogen activator; Rac 1, Rac 1 GTPase; values in the table are GAPDH-normalized, except Rac1 GTPase.

### 2.5. Immunohistochemistry Results

**Figure 6:**
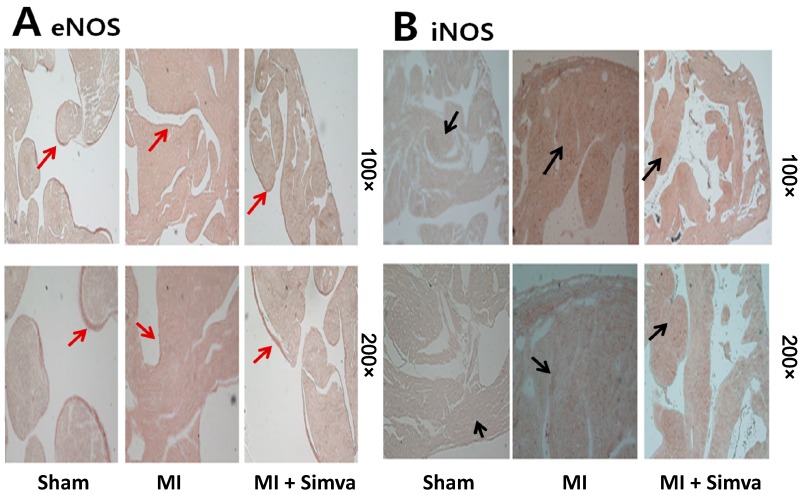
(**A**) Immunohistochemical staining showed that eNOS expression in the MI group was lower than the sham, and eNOS expression in the MI + simvastatin group was higher than the MI group (endothelial line, red arrows); and (**B**) iNOS expression in the MI group was higher than the sham, and iNOS expression in the MI + simvastatin group was lower than the MI group (cytoplasmic brown area, black arrows).

Immunohistochemical staining for eNOS showed that most activity was located at the endocardial area in the sham group. Endocardial eNOS activity was lower in the MI group than in the control group, but the MI + simvastatin group had higher endocardial eNOS activity than the MI group (Figure 6A). Immunohistochemical staining for iNOS revealed little activity in the sham group, but myocardial iNOS activity was higher in the MI group. The MI + simvastatin group had lower iNOS activity in the myocardium than the MI group (Figure 6B).

### 2.6. Thrombogenicity and Rac 1 Activity

The levels of TM, TFPI, tPA and eNOS proteins in LA tissue were significantly decreased in the MI group than in the sham group (*p* < 0.05). These changes were improved in the MI + simvastatin group compared to the MI group (*p* < 0.05) (Figure 5 and Table 2). All results were normalized to GAPDH data concomitantly obtained from the same samples. Cardiac Rac1 expression was strongly increased in the MI group, and the MI + simvastatin group showed significantly reduced Rac1 expression (*p* < 0.05) (Figure 5C and Table 2).

This study demonstrated increased Rac1, *I_NCX_* and iNOS expression, reduced SERCA and eNOS expression and activation of endothelial thromboprotective markers in a murine ischemic HF model. These findings were associated with an increased inducibility and duration of AF, and these changes were attenuated with simvastatin treatment, suggesting that the reduction of oxidative stress may be one of the mechanisms responsible for the antiarrhythmic effects of simvastatin in ischemic HF.

Recently, there have been several reports that have demonstrated an association between AF and the inflammatory process [2,3,4,5], and it has been suggested that statins may reduce vascular inflammation and the incidence of AF [12] due to antioxidant and anti-inflammatory effects [13,14]. Randomized clinical trials examining the efficacy of statins in HF (GISSI-HF and CORONA) did not show a benefit in mortality for patients with HF randomized to receive statins; however, the antiarrhythmic effects of statins in ischemic HF have not been investigated until now.

Our study addressed two mechanisms underlying the arrhythmias seen in ischemic HF. The first mechanism is reentry, which is associated with an increased amount of tissue fibrosis [22]. LA fibrosis is an important contributor to the AF seen in ischemic HF, and we found that simvastatin treatment decreased the LA fibrosis induced by the MI with which AF could be induced. Simvastatin also induced a regression of the cardiac hypertrophy and fibrosis and improved cardiac function in a transgenic rabbit model of human hypertrophic cardiomyopathy [23]. These results are consistent with the idea that a reduction in fibrosis may contribute to the antiarrhythmic effects of simvastatin. The other important mechanism of arrhythmias in HF is triggered activity [19], and Fenelon *et al.* [24] demonstrated that the majority of AF episodes had a focal mechanism by performing biatrial mapping in dogs with CHF. Simvastatin attenuated the propensity toward AF in the dog atrial tachycardia model [25], and it attenuated tachypacing-induced remodeling effects, including downregulation of the L-type Ca^2+^ channel alpha subunit expression and shortening of the effective refractory period (ERP).

NADPH oxidase activity is critical for generating oxidative stress, and activation of Rac1 GTPase might contribute to the pathogenesis of AF via activation of superoxide, producing NADPH oxidase [21]. Small GTPase Rac1 regulates certain subfamilies of NADPH oxidase, namely NOX1 and NOX2 [26,27]. NOX2 is upregulated only in patients with paroxysmal/persistent AF and is responsible for the overproduction of isoprostanes [28]. Statins block the isoprenylation and activation of small GTP-binding proteins members, such as the Ras and Rho family. An animal model has demonstrated that statins attenuate NADPH oxidase-induced superoxide production through the inhibition of Rac1 [29,30]. Based on these data, our result for the simvastatin-induced inhibition of Rac1 may decrease NOX2, which may reduce the risk of paroxysmal AF. However, in the case of permanent AF, this impact would be attenuated, because upregulation of atrial NADPH oxidases is an early, but transient event in the natural history of AF [30]. Moreover, our results showed that the increased Rac1 activity was associated with decreased atrial endothelial thromboprotective markers, as well as increased AF duration in ischemic HF rats. In addition to the inhibition of Rac1, the effect of statins on the isoprenylation of other proteins, as well as the anti-inflammatory effects of simvastatin is likely to contribute to the observed effects. Therefore, simvastatin might be useful in preventing thrombosis-related complications of AF.

Because changes in the NO synthase system were reported in a pig model of AF [4], the alteration in NO synthesis is thought to be an important contributor to AF-induced LA remodeling. In myocardial tissue, eNOS is constitutively expressed in the myocytes, endothelial cells and endocardial endothelium, while iNOS is induced after stimulation with lipopolysaccharide and cytokines in multiple cardiac cells, including myocytes, vascular endothelial and smooth muscle cells and inflammatory cells [31]. Because iNOS is expressed in pathological conditions, such as septic shock and HF [32], increased NO production in HF [33] is in part due to this increased iNOS and neuronal NO synthase (nNOS) expression [34]. In this study, we demonstrated decreased eNOS and increased iNOS expression in the LA tissue of the rat model of ischemic HF. Decreased eNOS is associated with an increased thrombogenicity in atrial tissue during HF, as evidenced by an increase in plasminogen activator inhibitor-1 (PAI-1) expression in the LA in a dog AF model [17]. These results support the idea that changes in eNOS expression contribute to tachypacing-induced atrial remodeling [4,32]. Interestingly, we also observed increased levels of iNOS in our study, which may also play a role in HF-induced LA remodeling in AF. A recent study demonstrated the relationship between superoxide and AF [35,36], and iNOS is associated with inflammation and superoxide synthesis. Increased iNOS suggests increased superoxide production in the LA tissue during HF, and statins have pleiotropic effects, including antioxidant and anti-inflammatory effects. In this study, we demonstrated that simvastatin prevented iNOS production in LA tissue during HF, and iNOS reduction may contribute to the antiarrhythmic effects of statins in HF.

This study also demonstrated increased production of *I*_NCX_ and decreased production of SERCA. HF increased the expression of *I*_NCX_, an important carrier of post-repolarization transient inward currents that cause delays after depolarizations (DADs). DADs can induce triggered activity, and there is evidence for the role of DADs in atrial tachyarrhythmias in canine CHF models [37,38]. Therefore, increased *I*_NCX_ expression in our study correlated with an increased susceptibility to AF in our rat model of HF, and decreased *I*_NCX_ expression in simvastatin-treated rats is consistent with the concept of statins as anti-arrhythmic agents. SERCA, which plays a major role in the contraction-relaxation cycle [39], was reduced in our HF group. Reduced SERCA expression is also an important contributor to HF-induced LA remodeling, and SERCA expression was significantly higher in animals treated with simvastatin.

## 3. Experimental Section

### 3.1. Animals Models

Animal procedures were performed according to National Institutes of Health guidelines. The study protocol was approved by the Ethical Committee of Kosin University School of Medicine (Kosin 13-01, 6 February 2013). Seven- to eight-week-old healthy male Sprague–Dawley rats weighing 250–300 grams were obtained from the Daehan Biolink Inc. (Chungbuk, Korea). Animals were housed in plastic cages (five animals each) and fed with a normal sodium diet and given access to tap water. They were maintained under standard laboratory conditions (controlled temperature of 21–24 °C, controlled light cycle consisting of alternating 12 h periods of light and darkness) during all experiments. An experimental rat model of post-MI HF was made from the ligation of the left anterior descending coronary artery (LAD), according to our previous study [37]. The rats were randomized into untreated MI and MI + simvastatin (2 mg/kg/day) groups after 2 days and observed for 10 weeks. The drugs were added to the drinking water, with careful monitoring of water consumption and body weight to ensure a proper drug dosage. Sham operated rats were used as a control group. The perioperative mortality was around 40% in the rats submitted to coronary artery ligation. The study was not financially funded by the pharmaceutical industry; however, simvastatin was generously provided by Merck Pharmaceuticals. Simvastatin was chosen because of the great lipophilicity, and it suppressed tachypacing-induced shortening of atrial refractoriness [25].

### 3.2. Echocardiogram

Echocardiography was performed in animals just before they were sacrificed. Animals were anesthetized by an intraperitoneal injection of ketamine (25 mg/kg) with xylazine (5 mg/kg). The chest was shaved, and the animals were placed on a warming pad to maintain normal body temperature. Transthoracic echocardiography was performed using a 12-MHz phased-array probe, Acuson Sequoia C512 (Mountain View, CA, USA). LV end-diastolic septal and posterior wall thickness (IVSd and PWTd, respectively), LV end diastolic dimension (LVEDD) and LV end systolic dimension (LVESD) were measured. The LV ejection fraction (EF) and fractional shortening (FS) were calculated according to the following formula: FS (%) = (LVEDD − LVESD)/LVEDD, EF (%) = (LVEDD2 − LVESD2)/LVEDD2 × 100%. Left atrial (LA) and aorta diameters were measured from the M-mode recordings in a modified parasternal long axis view.

### 3.3. Electrophysiological Study

The AF induction studies were performed in an open-chest state with a bipolar lead attached to the right atrial appendage. AF was induced by right atrial burst pacing (four times the pacing threshold for 30 s at 600 bpms). The mean duration of AF in each rat was determined on the basis of ten inductions. AF was induced ten times if the AF duration was ≤20 min and five times if the AF lasted between 20 and 30 min. AF that lasted >30 min was considered to be persistent, and the mean AF duration was counted as 30 min.

### 3.4. Quantification of Fibrosis

After completion of the electrophysiological study, the heart was rapidly removed and weighed, and the heart to body weight ratio was calculated. The hearts were immersed in a 10% buffered formalin solution and then embedded in paraffin. To identify possible interstitial fibrosis, Masson-Trichrome staining was carried out in a NexES Special Stainer (Ventana Medical Systems, Tucson, AR, USA). The amount of fibrosis in the LA, which was expressed as a percentage of perimuscular interstitial blue area except muscle for each rat, was quantified with the acquisition software Image-Pro Plus version 5.1 (Media Cybernetics, Maryland, GA, USA).

### 3.5. Western Blot

About 25 mg of rat LA tissue were homogenized in 1 mL of a modified tonic sucrose solution containing 0.3 mol/L sucrose, 10 mmol/L imidazole, 10 mmol/L sodium metabisulfite, 1 mmol/L DTT and 0.3 mmol/L PMSF. After centrifugation at 1300× *g*, 50 μg of protein for the iNOS and Na^+^–Ca^2+^ exchanger (INCX), thrombomodulin (TM), tissue factor pathway inhibitor (TFPI), tissue plasminogen activator (tPA) and 25 μg of protein for the eNOS and SERCA were loaded into 10% SDS-polyacrylamide gel electrophoresis gel and transferred to a PVDF membrane (Pierce, Rockford, IL, USA). The blocked membranes were then incubated with the antibodies to eNOS, iNOS (BD Transduction Laboratories, San Jose, CA, USA), TFPI, TM (American Diagnostica Inc., Stamford, CT, USA) and NCX (Affinity BioReagents, Golden, CO, USA). Horseradish peroxidase (HRP)-conjugated IgG antibody (Santa Cruz Biotechnology, Santa Cruz, CA, USA) was used as the secondary antibody. The immunoblot was visualized using chemiluminescent reagent as recommended by the SuperSignal West Pico Chemiluminescent Substrate kit (Pierce, Rockford, IL, USA). As an internal control, this membrane was re-blotted with anti-GAPDH antibody.

### 3.6. Rac1 GST-p21-Activated Kinase Pull-Down Assay

Tissue was homogenized and resuspended in magnesium-containing lysis buffer (25 mmol/L HEPES (pH 7.5); 150 mmol/L sodium chloride; 1% Igepal CA-630; 10% glycerol; 25 mmol/L sodium fluoride; 10 mmol/L magnesium chloride; 1 mmol/L EDTA; 1 mmol/L sodium orthovanadate; 10 µg/mL leupeptin; 10 µg/mL aprotinin) and centrifuged at 1000 rpm for 5 min at 4 °C. Equal amounts of supernatant protein were incubated with 10 µL of agarose-labeled p21-activated kinase (PAK)-1 fusion protein at 4 °C for 60 min. Beads were washed 3-times with magnesium-containing lysis buffer, eluted in Laemmli buffer (60 mmol/L Tris (pH 6.8), 2% sodium dodecyl sulfate, 10% glycerin, 0.1% bromophenol blue) and analyzed for bound Rac1 in relation to total Rac1 content by western blotting.

### 3.7. Immunohistochemistry for eNOS and iNOS

Sections of left atrial tissue were immunostained with eNOS and iNOS antibody using a horseradish peroxidase-streptavidin-biotin method (UltraVision LP Detection System, Lab Vision Corporation, Suffolk, UK) according to the manufacturer’s recommendation. Briefly, formalin-fixed, paraffin-embedded tissue was prepared using conventional histological methods. Serial sections (6 μm) were cut from each paraffin block. Samples were immersed in 10% neutral buffered formalin for 30 min, permeabilized with 0.1% Triton X-100 for 15 min, and antigen retrieval was achieved by heating in 0.01 M citric acid, pH 6.0, for 20 min. Nonspecific binding was blocked with normal goat serum for 15 minutes. The sections were incubated with anti-eNOS antibody (Santa Cruz, CA, USA) or anti-iNOS antibody (Santa Cruz, CA, USA) at a 1:50 dilution in PBS. After washing with PBS twice for 5 min each time, incubation was performed for 1 h at room temperature with biotinylated rabbit anti-rat IgG (1:50) in PBS. Next, the slides were incubated with streptavidin-peroxidase conjugate for 15 min. Peroxidase was visualized by the addition of 3-amino-9-ethyl carbazole (ACE) and hydrogen peroxide. Negative controls were carried out under the same conditions using rat IgG instead of eNOS or iNOS antibody.

### 3.8. Statistical Analysis

We used the resource equation method [40], because it was not possible to assume the effect size, to get an idea about the standard deviation, as no previous findings are available and multiple endpoints are measured. According to this method, a value E (total number of animals − total number of groups) is measured, which is nothing but the degree of freedom of analysis of variance (ANOVA). Any sample size that keeps *E* between 10 and 20 should be considered as adequate. Because we want to see the effect of simvastatin, we made three groups with 10 rats each considering measurement failure, and *E* was 27, which is more than necessary. The statistical analysis for each parameter studied was carried out with ANOVA. If the ANOVA tests were significant, inter-group comparisons were performed using the Mann–Whitney test. *p*-values less than 0.05 were considered to be statistically significant. All results are expressed as the mean ± standard deviation (SD). For the statistical analysis, SPSS version 12.0 (SPSS Inc., Chicago, IL, USA) was used.

## 4. Conclusions

Our observation suggests that the small G protein Rac1 GTPase is upregulated in the ischemic HF model with reduced eNOS, activation of endothelial thromboprotective markers, induction of fibrosis, and increased susceptibility for of AF induction. Simvastatin treatment, which reduces Rac1 activity, was associated with a reduced inducibility of AF, LA fibrosis and thrombogenicity. These results provide a simvastatin-preventing effect of AF in ischemic HF in rats and may be an interesting new approach for preventing arrhythmias associated with ischemic HF-induced LA remodeling in humans.

A limitation of the study is that the model of AF in ischemic HF was induced by pacing. However, classical cardiovascular risk factors influence the genesis, perpetuation, oxidative stress and atherothromboembolism of AF in humans [41]. The dosage of the simvastatin in our study (2 mg/kg/day) was equal to that used in some experimental studies and smaller than that in others, but it was somewhat higher than that used commonly for hypercholesterolemia (0.3 to 1 mg/kg) [25]. It still remains to be determined whether clinically-used doses of simvastatin are able to prevent HF-induced AF in humans.
